# The role of viscoelastic tests in the diagnosis of sepsis-induced coagulopathy (SIC)

**DOI:** 10.1186/s13613-025-01442-2

**Published:** 2025-01-28

**Authors:** Toshiaki Iba, Julie Helms, Jerrold H. Levy

**Affiliations:** 1 Department of Emergency and Disaster Medicine, Juntendo University Graduate School of Medicine, 2-1-1 Hongo Bunkyo-ku, Tokyo, 113-8421 Japan; 2 Medical Intensive Care Unit - NHC, Strasbourg University (UNISTRA), Strasbourg University Hospital, INSERM (French National Institute of Health and Medical Research), UMR 1260, Regenerative Nanomedicine (RNM), FMTS, Strasbourg, France; 3 Department of Anesthesiology, Critical Care, and Surgery, Duke University School of Medicine, Durham, NC USA

We thank Dr. Yum Ji for his interest in our article. She suggested viscoelastic tests (VETs), including thromboelastography (TEG) and rotational thromboelastometry (ROTEM) may be valuable for diagnosing sepsis-associated disseminated intravascular coagulation (DIC) [1]. We agree that these tests provide real-time evaluation of the clotting process, from clot formation to stabilization and dissolution, which are dynamically altered in sepsis and can distinguish between hypercoagulable and hypocoagulable states [2]. However, unlike detecting DIC, the utility of VETs in diagnosing sepsis-induced coagulopathy (SIC) may be limited. SIC represents the early phase of DIC caused by sepsis, and detecting DIC’s decompensated (hypocoagulable) phase is not a primary requirement.

One of the benefits of VETs is the ability to diagnose hyperfibrinolysis, which is not advantageous in SIC. This capability is particularly clinically useful in specific conditions such as the hypercoagulable type DIC or trauma-induced coagulopathy (TIC), where abnormalities in the fibrinolytic system play a critical role and require potential therapeutic interventions with tranexamic acid. Conventional biomarker testing evaluating D-dimer and plasmin-α2-antiplasmin complex (PAP) provide indirect evidence of hyperfibrinolysis, while VETs visualize the dynamic process of clot formation and lysis, offering more rapid diagnostic insights, especially in bleeding associated with TIC. In addition, VETs are particularly useful for guiding antifibrinolytic therapy (e.g., tranexamic acid) based on the presence of hyperfibrinolysis. For instance, in trauma patients or those with obstetric hemorrhage, identifying hyperfibrinolysis facilitates appropriate treatment with major bleeding. Limitations of VET include availability as many hospitals may not have this test, and their measurements lack standardization between the devices and procedures.

Given the high prevalence of SIC, an ideal screening method should be simple, cost-effective, and universally accessible [3]. For these reasons, we recommend prothrombin time and platelet counts as the components of SIC diagnostic criteria. Besides clinical use, SIC was designed to facilitate clinical trials in DIC [4]. For the success of the trials, appropriate screening of the candidates and timely initiation of the treatment are essential, and screening with standardized coagulation tests is favorable. In contrast to their use in SIC, VETs offer advantages over standard coagulation tests in TIC, including rapid assessment, comprehensive evaluation of clot dynamics, point-of-care availability, and facilitate goal-directed therapy [5]. However, although many studies highlight associations between VET measurements and early coagulopathies, their predictive performance is not consistently superior to conventional tests. VETs, with their rapid, point-of-care nature, enable quicker decision-making in time-sensitive situations and are particularly suitable for detecting TIC and other hypocoagulable forms of DIC. In contrast to TIC, SIC is characterized as a hypercoagulable type of DIC that does not require urgent diagnosis as much as TIC but is notably prevalent. Commonly available traditional assays with less cost are favorable for diagnosing this type of DIC.

Finally, it is important to note that in septic patients, overt DIC patients progress through SIC during the course of the disease. As a result, overt DIC patients should be identified and diagnosed as SIC at an earlier stage. Current studies and inclusion criteria for management are not routinely based on VET values. Therefore, it may not be adequate to replace any component of overt DIC criteria with VETs. However, we agree with the author that further research is needed to apply VET to and explore its potential value in clinical management.

## Data Availability

Not applicable.

## Abbreviations

VETs: viscoelastic tests
TEG: thromboelastography
ROTEM: rotational thromboelastometry
DIC: disseminated intravascular coagulation
SIC: sepsis-induced coagulopathy
TIC: trauma-induced coagulopathy
PAP: plasmin-α_2_-antiplasmin complex
